# Identifying Pediatric Long COVID: Comparing an EHR Algorithm to Manual Review

**DOI:** 10.1055/a-2702-1574

**Published:** 2025-10-24

**Authors:** Morgan Botdorf, Kimberley Dickinson, Vitaly Lorman, Hanieh Razzaghi, Nicole Marchesani, Suchitra Rao, Colin Rogerson, Miranda Higginbotham, Asuncion Mejias, Daria Salyakina, Deepika Thacker, Dima Dandachi, Dimitri A. Christakis, Emily Taylor, Hayden T. Schwenk, Hiroki Morizono, Jonathan D. Cogen, Nathan M. Pajor, Ravi Jhaveri, Christopher B. Forrest, L. Charles Bailey, on behalf of the RECOVER Consortium

**Affiliations:** 1 Applied Clinical Research Center, Children's Hospital of Philadelphia, Philadelphia, Pennsylvania, United States; 2 Department of Pediatrics, University of Colorado School of Medicine and Children's Hospital Colorado, Denver, Colorado, United States; 3 Division of Critical Care, Department of Pediatrics, Indiana University School of Medicine, Indianapolis, Indiana, United States; 4 Division of Infectious Diseases, Department of Pediatrics, Nationwide Children's Hospital and The Ohio State University, Columbus, Ohio, United States; 5 Center for Precision Medicine, Nicklaus Children's Hospital, Miami, Florida, United States; 6 Nemours Cardiac Center, Alfred I. duPont Hospital for Children, Wilmington, Delaware, United States; 7 Division of Infectious Diseases, Department of Medicine, University of Missouri-Columbia, Columbia, Missouri, United States; 8 Center for Child Health, Behavior and Development, Seattle Children's Research Institute, Seattle, Washington, United States; 9 New York University Grossman School of Medicine, RECOVER Patient, Caregiver, or Community Representative, New York, New York, United States; 10 Division of Pediatric Infectious Diseases, Stanford School of Medicine, Palo Alto, California, United States; 11 Center for Genetic Medicine Research, Children's National Hospital, Washington, District of Columbia, United States; 12 Division of Pulmonary and Sleep Medicine, Department of Pediatrics, Seattle Children's Hospital, University of Washington, Seattle, Washington, United States; 13 Division of Pulmonary Medicine, Cincinnati Children's Hospital Medical Center and University of Cincinnati College of Medicine, Cincinnati, Ohio, United States; 14 Division of Infectious Diseases, Ann & Robert H. Lurie Children's Hospital of Chicago, Chicago, Illinois, United States

**Keywords:** long COVID, COVID-19, pediatrics, electronic health records, computable phenotype, PEDSnet

## Abstract

**Background:**

Long COVID, characterized by persistent or recurring symptoms post-COVID-19 infection, poses challenges for pediatric care and research due to the lack of a standardized clinical definition. Adult-focused phenotypes do not translate well to children, given developmental and physiological differences, and pediatric-specific phenotypes have not been compared with chart review.

**Objective:**

This study introduces and evaluates a pediatric-specific rule-based computable phenotype (CP) to identify long COVID using electronic health record data. We compare its performance to manual chart review.

**Methods:**

We applied the CP, composed of diagnostic codes empirically associated with long COVID, to 339,467 pediatric patients with SARS-CoV-2 infection in the RECOVER PCORnet EHR database. The CP identified 31,781 patients with long COVID. Clinicians conducted chart reviews on a subset of patients across 16 hospital systems to assess performance. We qualitatively reviewed discordant cases to understand differences between CP and clinician identification.

**Results:**

Among the 651 reviewed patients (339 females, *M*_age_ = 10.10 years), the CP showed moderate agreement with clinician identification (accuracy = 0.62, positive predictive value [PPV] = 0.49, negative predictive value [NPV] = 0.75, sensitivity = 0.52, specificity = 0.84). Performance was largely consistent across age and dominant variant but varied by symptom cluster count. Most discrepancies between the CP and chart review occurred when the CP identified a case, but the clinician did not, often because clinicians attributed symptoms to preexisting conditions (73%). When clinicians identified cases missed by the CP, they often used broader symptom or timing criteria (69%). Model performance improved when the CP accounted for preexisting conditions (accuracy = 0.71, PPV = 0.65, NPV = 0.74, sensitivity = 0.59, specificity = 0.79).

**Conclusion:**

This study presents a CP for pediatric long COVID. While agreement with manual review was moderate, most discrepancies were explained by differences in interpreting symptoms when patients had preexisting conditions. Accounting for these conditions improved accuracy and highlights the need for a consensus definition. These findings support the development of reliable, scalable tools for pediatric long COVID research.

## Background and Significance

Long COVID, also known as postacute sequelae of SARS-CoV-2 infection (PASC), is a significant health concern characterized by ongoing, relapsing, or new symptoms occurring four or more weeks after the acute infection. 1 While some postviral syndromes, such as chronic fatigue syndrome, are well-documented in children, 2 3 less is known about the clinical manifestations of long COVID in pediatric patients. Studies suggest that between 1 and 20% of children who contract COVID-19 may develop long COVID symptoms, though these estimates vary widely depending on study design, sample size, and population characteristics. 4 5 6 7

Long COVID symptoms in children can range from fatigue and headache to loss of taste and smell and chest pain. 5 8 9 10 11 12 Although rare, long COVID has also been associated with myocarditis, postural tachycardia syndrome (POTS), and myalgic encephalomyelitis/chronic fatigue syndrome, among other conditions. 13 Some conditions, like multisystem inflammatory syndrome (MIS-C), are clearly attributable to a SARS-CoV-2 infection, but much remains to be understood about others. 14 15 The variability in long COVID symptoms complicates clear and consistent diagnosis, which typically relies on clinical evaluation of the patient's symptoms and their COVID-19 history. This underscores the need for further research to establish clear diagnostic standards and improved care for affected children.

Identifying children with long COVID in research studies is crucial to better understand this syndrome and ensure timely detection and treatment. However, like diagnosing long COVID in a clinical setting, this task is challenging due to the inconsistency and heterogeneity of symptoms. To address this challenge, researchers have used large observational cohort studies that use repositories of electronic health record (EHR) data to identify patients. 9 11 12 16 17 Typically, these studies rely on EHR-based diagnosis codes, 16 17 such as the ICD-10-CM U09.9 code, introduced in October 2021. 18 19 While this code allows clinicians to assign a post-COVID diagnosis, its utilization is inconsistent across patients and healthcare settings 17 and relying solely on this code poses a misclassification risk due to the variety of long COVID symptoms.

To improve identification of patients with long COVID, researchers have used computable phenotyping, which involves developing a set of rules to identify patients with a disorder. While phenotypes have been developed for use in adult populations, 20 they cannot be directly translated to pediatric populations due to physiological, developmental, and communication differences between adults and children. These differences underscore the need for phenotyping approaches tailored to and validated in pediatric populations.

Limited long COVID phenotypes for pediatric patients have been developed using machine-learning approaches that leverage large numbers of clinical features. For example, in a recent pediatric study, a machine learning algorithm demonstrated high precision in classifying general and MIS-C-specific forms of PASC in pediatric patients. 21 These approaches show promise, but their reliance on large, labeled training datasets limits generalizability. Furthermore, these algorithms lack validation with clinical adjudication and an exploration of reasons for discordance.

To address this gap, we developed a rule-based computable phenotype (CP) to identify pediatric long COVID using the RECOVER PCORnet EHR database, a large multi-site EHR dataset, and compared its performance against manual chart review. Importantly, our phenotype incorporates symptom clusters more common in those with a COVID-19 infection, rather than relying solely on a diagnosis code for long COVID. We qualitatively reviewed discordant cases to assess limitations in automated and manual classification approaches. This study represents a significant step in transparent, reproducible, and scalable phenotyping of long COVID, informed by clinical insight, which is critical in the absence of a standardized pediatric definition. Given that long COVID can impose a substantial burden on children and their families, leading to missed school and the need for service referrals, 22 23 improved detection of long COVID supports the development of effective tools for research and treatment.

## Methods

### Data Source

This retrospective study is part of the NIH Researching COVID to Enhance Recovery (RECOVER) Initiative, which seeks to understand, treat, and prevent the postacute sequelae of SARS-CoV-2 infection. 24 It uses data from the RECOVER PCORnet EHR database, which includes more than 9 million children from 40 hospital systems across the United States who were tested, diagnosed, or vaccinated for COVID-19 between 2019 and December 2022. Institutional Review Board (IRB) approval was obtained under Biomedical Research Alliance of New York (BRANY) protocol no.: 21–08–508. BRANY waived the need for consent and HIPAA authorization.

### Study Population

**Fig. 1:**
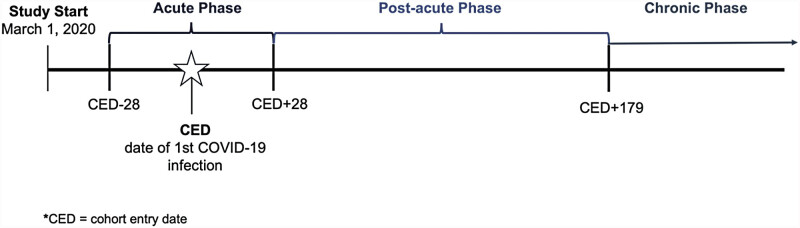
Study timeline.

Inclusion criteria for our pediatric sample were as follows: (1) SARS-CoV-2 infection confirmed via clinical diagnosis or PCR, antigen, or qualifying serology test 25 between March 2020 and December 2022; (2) age less than 21 years at first COVID-19 infection; and (3) at least two encounters with the healthcare system (at least one in-person or telehealth). The encounters were required to occur between 28 and 179 days after the initial COVID-19 infection to ensure adequate follow-up for assessing long COVID outcomes. We defined clinically meaningful time periods surrounding the initial COVID-19 infection as shown in Fig. 1 . The postacute phase (days 28–179 postinfection) was the primary focus of analyses. For patients with a specific COVID-19 diagnosis or viral test, the initial infection date was the date of diagnosis or test. For patients with diagnoses indicating “history of” or “complication of” COVID-19 or with a positive serology test, we used 28 days prior to the earliest diagnosis or test evidence of COVID-19 as a proxy for the initial infection date.

### Phenotype Classification

**Fig. 2:**
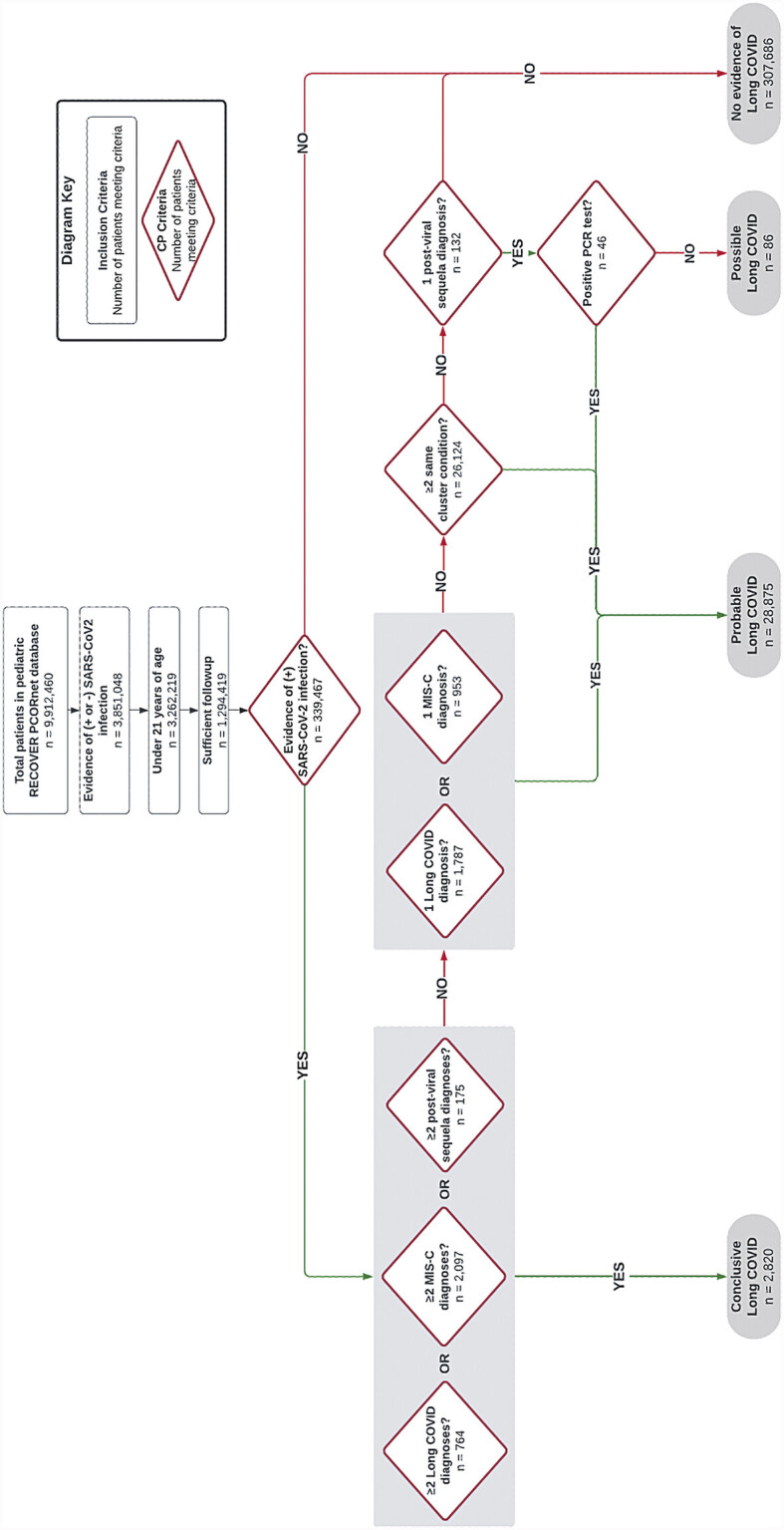
Flow chart depicting attrition and the algorithm used to identify patients with long COVID.

Fig. 2 depicts the steps applied to classify patients according to the certainty of having long COVID. Patients were identified as having “conclusive,” “probable,” or “possible” long COVID according to the algorithm, which used criteria documented in the EHR in the postacute period. Patients meeting any of these categories were labeled as “long COVID evidence,” and all others were labeled as “no long COVID evidence.” Additional details related to the phenotype classification are included in Supplementary Methods (available in the online version only).

### Chart Review Sampling

**Fig. 3:**
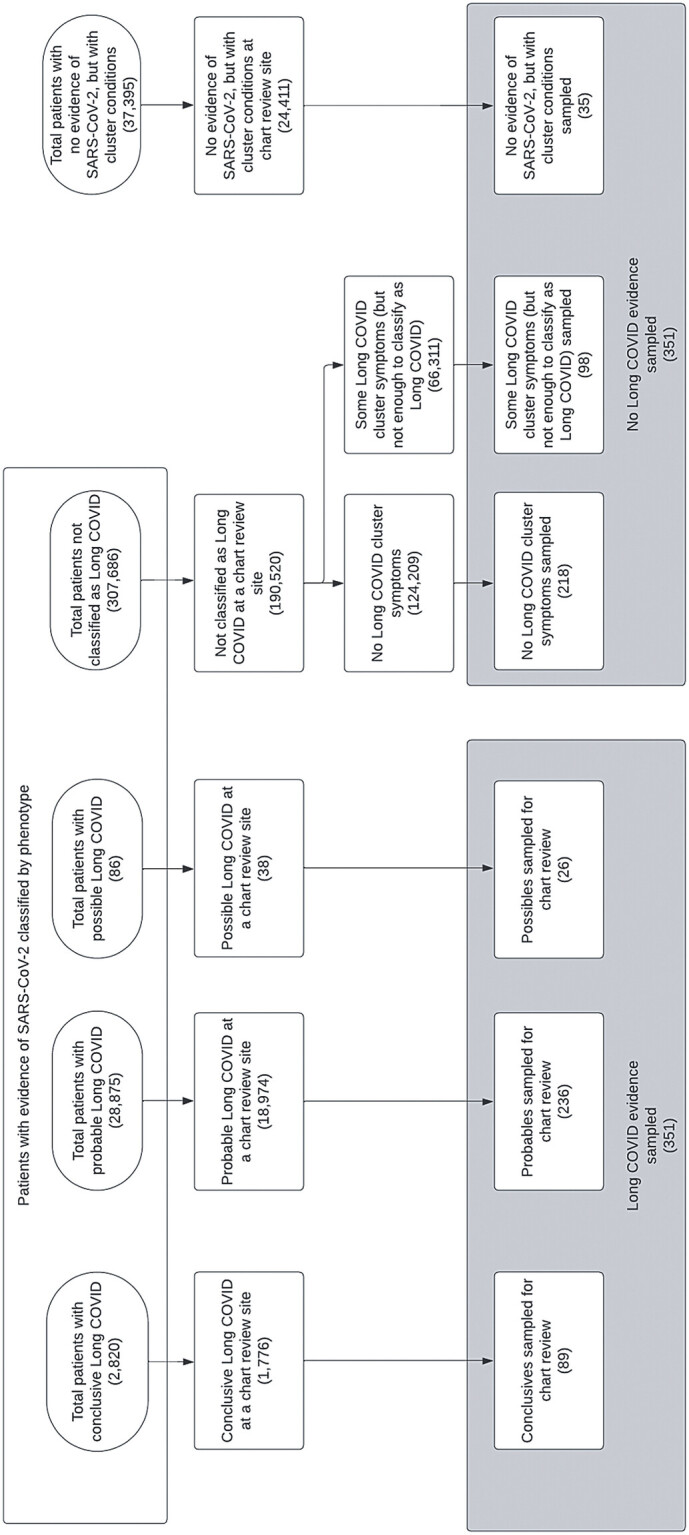
Sampling strategy for the chart review cohort. The numbers reported in parentheses represent sample sizes.

A manual chart review was performed on a subset of 702 patients at 16 healthcare institutions, which are listed in Supplementary Table S1 (available in the online version only). Patients were split between the long COVID evidence and no long COVID evidence groups using 1:1 exact matching. The sampling strategy is laid out in Fig. 3 , and detailed information is provided in Supplementary Methods (available in the online version only).

### Chart Review Procedure

Clinical research teams from each participating institution conducted chart reviews using a REDCap 26 (Research Electronic Data Capture) instrument with questions including information on COVID-19 diagnoses and testing, demographics, COVID-19 prevention and treatment strategies, vaccines, functional outcomes, and conditions post-COVID-19 captured in the patient's medical record, both in notes and structured fields. Each site had between 1 and 5 reviewers for a total of 44 reviewers. The case report form is included in Supplementary File 2 (available in the online version only).

After the initial review was completed by a research team member, a clinician at each site conducted a secondary review. Using the chart review data, the clinician answered questions regarding their confidence in identifying long COVID in the patient. To simplify the assessment of the CP's performance compared with chart review, patients were grouped into four overlapping categories: CP-positive, CP-negative, clinician review-positive (CR-positive), and clinician review-negative (CR-negative). Detailed information related to the chart review procedure is included in Supplementary Methods 27 (available in the online version only).

We compared sociodemographic characteristics between patients classified with and without long COVID using standardized mean differences (SMDs). An SMD of < 0.2 was considered a good balance.

### Performance Assessment

We evaluated the performance of the CP across various key metrics, including sensitivity, specificity, positive predictive value (PPV), and negative predictive value (NPV). Additionally, we examined the accuracy and F1 score of the phenotype. Accuracy assesses the proportion of CR-positive patients who were also CP-positive. The F1 score combines precision and recall, providing insight into the overall effectiveness of the phenotype. All analyses were conducted using R version 4.1.2 28 (2021–11–01).

Next, we assessed whether performance differed by age (i.e., under vs. over 12 years old; see Supplementary Methods , available in the online version only), infection era (i.e., α, delta, omicron; see Supplementary Table S2 , available in the online version only), and number of symptom clusters through stratified analyses.

We then identified discrepancies between the phenotype and the chart review identification of long COVID. To understand the reasons behind these discrepancies, we reviewed the chart review form for each discordant patient, along with the clinician reviewer's explanation for assigning or not assigning long COVID. We then generated themes that accounted for the discrepancy and assigned those themes to the remaining cases.

### Sensitivity Analyses

We aimed to modify our algorithm based on the most common themes and perform a sensitivity analysis to assess whether the performance of our modified algorithm was superior to the original algorithm.

Given that long COVID symptoms could be intertwined with preexisting conditions, we identified patients with complex medical histories to describe the sample and investigate how medical complexity affected the performance of the phenotype. To do this, we used the more conservative version of the Pediatric Medical Complexity Algorithm 29 (PMCA), which classifies a patient as having a complex chronic disease if the patient has one diagnosis of a progressive or malignant condition or at least two diagnoses in at least two body systems in the 3 years prior to the SARS-CoV-2 infection.

Finally, we conducted an additional analysis including only patients classified as “conclusive” long COVID in both the CP-positive and CR-positive groups. All other cases were classified as negative. We then re-evaluated the performance of the phenotype using this stricter case definition to assess its accuracy when ambiguous cases were excluded.

## Results

### Sample Characteristics

Among 1,007,867 patients with confirmed SARS-CoV-2 infection, the CP detected long COVID in 31,781 individuals (3.2%). Demographic characteristics and descriptive statistics for the final analytic sample of 651 patients (318 CP-positive and 333 CP-negative) are summarized in Table 1 . Sociodemographic characteristics were similar among the CP-positive and CP-negative groups as indicated by SMDs less than 0.20. Demographic characteristics for the CR-positive and -negative groups are summarized in Supplementary Table S3 (available in the online version only) and discussed in Supplementary Results (available in the online version only).

**Table 1:** Demographics of children with and without long COVID based on the computable phenotype definition

	Overall ( *n* = 651)	CP-detected long COVID ( *n* = 318)	No CP-detected long COVID ( *n* = 333)	SMD
Approx. CED age (y)				
Mean (SD)	10.10 (6.30)	10.10 (6.32)	10.10 (6.28)	<0.001
Median [min, max]	10.9 [0, 21.0]	11.0 [0, 21.0]	10.3 [0.1, 21.0]	
Age group (y)				
<1	49 (7.5%)	24 (7.5%)	25 (7.5%)	0.023
1–4	146 (22.4%)	70 (22.0%)	76 (22.8%)	
5–9	107 (16.4%)	52 (16.4%)	55 (16.5%)	
10–15	198 (30.4%)	98 (30.8%)	100 (30.0%)	
16–20	151 (23.2%)	74 (23.3%)	77 (23.1%)	
Patient sex				
Male	314 (48.2%)	147 (46.2%)	167 (50.2%)	0.079
Female	337 (51.8%)	171 (53.8%)	166 (49.8%)	
Race				
Asian/Native Hawaiian/Pacific Islander	26 (4.0%)	12 (3.8%)	14 (4.2%)	0.073
Black	115 (17.7%)	57 (17.9%)	58 (17.4%)	
White	357 (54.8%)	175 (55.0%)	182 (54.7%)	
Multiracial	21 (3.2%)	12 (3.8%)	9 (2.7%)	
Other/unknown	132 (20.3%)	62 (19.5%)	70 (21.0%)	
Ethnicity				
Hispanic	177 (27.2%)	78 (24.5%)	99 (29.7%)	0.118
Non-Hispanic	424 (65.1%)	214 (67.3%)	210 (63.1%)	
Unknown	50 (7.7%)	26 (8.2%)	24 (7.2%)	
Payer a				
Private	282 (43.3%)	139 (43.7%)	143 (42.9%)	0.026
Public	258 (39.6%)	124 (39.0%)	134 (40.2%)	
Other/Unknown	111 (17.1%)	55 (17.3%)	56 (16.8%)	

Note: CP, computable phenotype; SMD, standardized mean difference.

a At the time of COVID-19 infection.

### Overall Performance of the Computable Phenotype

We evaluated the performance of the CP against chart review. Performance statistics are presented in Table 2 . The CP identified 156 of 239 CR-positive cases (sensitivity = 65%) and 250 of 412 CR-negative cases (specificity = 61%). A full cross-tabulation of the CP and CR classifications is shown in Supplementary Table S4 (available in the online version only), and a site-level performance breakdown is provided in Supplementary Table S5 (available in the online version only).

**Table 2:** Statistics comparing computable phenotype and clinician review identification of long COVID

	CR-positive ( *n* = 239)		CR-negative ( *n* = 412)			
CP-positive ( *n* = 318)	156		162			
CP-negative ( *n* = 333)	83		250			
Performance statistics a						
	Accuracy	Sensitivity	Specificity	PPV	NPV	F1
	0.624	0.653	0.607	0.491	0.751	0.560

Note: CP, computable phenotype; CR, chart review; NPV, negative predictive value; PPV, positive predictive value.

a Assessed as CP relative to CR.

### Subgroup Performance of the Computable Phenotype

Analyses assessing whether performance differed by selected variables, including age at infection, infection era, and number of symptom clusters, showed largely similar results across groups ( Supplementary Tables S6–S8 , available in the online version only). Performance was similar across age groups with limited improvement in those at least 12 years of age, compared against those less than 12 years of age, including higher accuracy (0.65 vs. 0.60), sensitivity (0.69 vs. 0.62), PPV (0.55 vs. 0.44), and F1 score (0.61 vs. 0.52; Supplementary Table S6 , available in the online version only).

There were some variations in performance by infection era ( Supplementary Table S7 , available in the online version only). Accuracy, PPV, and the F1 score were highest during the Delta era. The F1 score was lowest during the Ancestral and Alpha eras. Performance varied more based on the number of symptom clusters, with a higher sensitivity and F1 score for patients with three or more symptom clusters ( Supplementary Table S8 , available in the online version only). In those with no symptom clusters, there was high specificity, accuracy, and NPV, but low sensitivity.

### Assessment of Discordant Cases between the Computable Phenotype and Chart Review

**Fig. 4:**
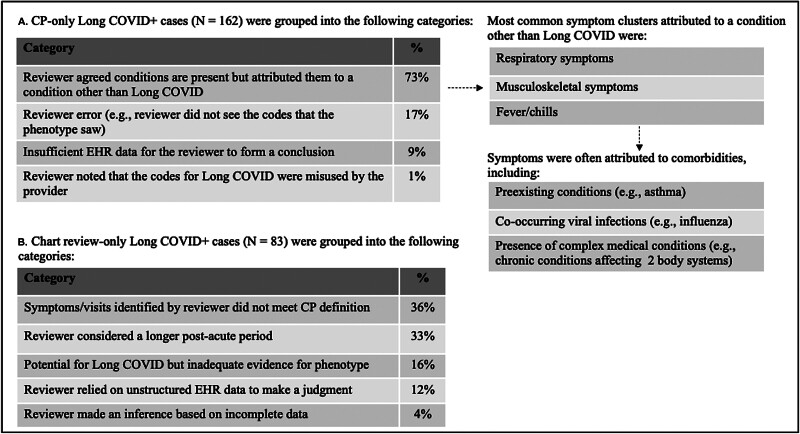
Qualitative review of ( **A** ) computable phenotype positive/chart review negative (CP+/CR−) long COVID patients and ( **B** ) chart review positive/computable phenotype negative (CR+/CP−) long COVID patients.

A review was conducted to assess reasons for disagreement between the two methods. The initial focus was on the most frequent discordant situation, where the phenotype identified long COVID, but the chart review did not (CP+/CR− cases, see Fig. 4 ). In most CP+/CR− cases, the clinician reviewer acknowledged the symptoms identified by the CP but attributed them to a non-COVID viral infection or preexisting condition ( Fig. 4A ). This was especially true for symptoms common to non-COVID respiratory infections or those present before and after COVID-19 infection. Less commonly, reviewers missed diagnostic codes that the phenotype saw, or the reviewer made a conclusion based on incomplete information.

An assessment of CR-positive/CP-negative (CR+/CP−) cases showed that the reviewer often considered symptoms, visits, and time frames that differed from our phenotype ( Fig. 4B ). For example, clinician reviewers considered symptoms beyond 180 days postinfection (up to 11 months in some cases) while the CP considered symptoms in 1 month to 180 days following infection. In addition, reviewers considered symptoms beyond those included in the phenotype definition, such as mental health symptoms (e.g., anxiety).

### Sensitivity Analyses

The review of CP+/CR− patients showed that comorbidities were a major factor contributing to discordance between the two methods. Given the difficulty of distinguishing symptoms due to underlying conditions versus long COVID, we performed a sensitivity analysis using a modified phenotype that accounted for preexisting conditions and comorbidities.

First, we assessed the phenotype's classification when nonincident diagnoses were excluded. In other words, we censored preexisting symptoms. Patients who met criteria for the CP with only preexisting symptom clusters that persisted after their COVID-19 diagnosis were labeled as having no evidence of long COVID ( *n* = 32 patients).

Second, given that symptoms like respiratory issues or fever are common in non-COVID-19 respiratory infections, we excluded any diagnoses from the respiratory or fever clusters that occurred within 2 weeks before or after a non-COVID-19 respiratory infection. Although SARS-CoV-2 may increase the risk of secondary infections, 30 31 the symptoms are typically caused by different agents. Therefore, we removed these infections as indicators of long COVID in our phenotype. This resulted in the reclassification of 31 patients from CP-positive to CP-negative who did not otherwise meet the criteria for long COVID.

Finally, it was difficult to attribute postacute symptoms to a COVID-19 diagnosis in patients with multiple chronic conditions. Therefore, we reclassified patients with complex chronic conditions according to the PMCA 29 as having no evidence of long COVID ( *n* = 67 patients).

### Performance Assessment After Modifications

Performance was reassessed after incorporating these alterations, and results are presented in Table 3 . The modified model showed higher accuracy (0.72 vs. 0.62), PPV (0.65 vs. 0.49), and specificity (0.84 vs. 0.61), but lower sensitivity (0.52 vs. 0.65). The NPV remained high (0.75), and the F1 score remained similar to the original model (0.58 vs. 0.56).

**Table 3:** Statistics comparing the modified computable phenotype and clinician review identification of long COVID

Modified						
	CR-positive ( *n* = 239)		CR-negative ( *n* = 412)			
Modified CP-positive ( *n* = 188)	123		65			
Modified CP-negative ( *n* = 463)	116		347			
No medically complex patients						
	CR-positive ( *n* = 210)		CR-negative ( *n* = 308)			
Modified CP-positive ( *n* = 188)	123		65			
Modified CP-negative ( *n* = 330)	87		243			
Performance statistics a						
	Accuracy	Sensitivity	Specificity	PPV	NPV	F1
Modified	0.722	0.515	0.842	0.654	0.749	0.576
No medically complex patients	0.707	0.586	0.789	0.654	0.736	0.618

Note: CP, computable phenotype; CR, chart review; NPV, negative predictive value; PPV, positive predictive value.

a Assessed as CP relative to CR.

Given the difficulty in adequately attributing diagnoses to long COVID in patients with complex medical histories, we also completed a sensitivity analysis excluding these patients from the sample, and performance was reassessed ( Table 3 ). Results showed similar performance to the modified model, but a slightly higher F1 score (0.62 vs. 0.58).

Results from the sensitivity analysis focused on CP- and CR-conclusive cases are reported in Supplementary Results (available in the online version only, Supplementary Table S9 , available in the online version only).

## Discussion

We evaluated a rule-based CP for identifying pediatric patients with long COVID in a large EHR database and compared it to clinician chart review. The two methods showed moderate agreement, with discrepancies largely associated with underlying comorbidities and non-COVID respiratory infections.

The original phenotype was moderately sensitive as it detected most, but not all, CR-positive cases. However, precision was lower, as indicated by the lower PPV with a high number of cases flagged as positive by the CP but not by the chart review. The CP was more reliable at ruling out patients without long COVID, reflected in a higher specificity and NPV. Across models, there was a consistent tradeoff between sensitivity and specificity with moderate F1 scores.

### Improvements through Phenotype Refinement and Subgroup Performance

Restricting the phenotype to incident diagnoses and excluding non-COVID respiratory infections improved accuracy, specificity, and PPV, but reduced sensitivity. Excluding medically complex patients resulted in the most balanced tradeoff between precision and recall with a higher F1 score than prior models. Removing these patients likely reduced ambiguity in symptom attribution and suggests that the CP may be less reliable in medically complex populations. Finally, our sensitivity analysis showed that performance also improved when the CP- and CR-positive groups were limited to conclusive cases. Sensitivity and specificity increased, likely due to more uniform and fewer ambiguous cases. Although fewer CP-predicted positives were confirmed by chart review, the CP was more reliable at ruling out long COVID.

When assessing performance by subgroup, the phenotype performed similarly across age groups and was most effective during the Delta era, potentially reflecting changes in clinical documentation or disease severity over time. As the number of symptom clusters increased, sensitivity and the F1 score improved, but specificity decreased, highlighting the balance between identifying more true cases and reducing false positives.

### Factors Contributing to Disagreement Between the CP and Chart Review

Chart review, while treated as the reference standard, is inherently subjective. This subjectivity is amplified in the case of long COVID, a condition that remains poorly defined, especially in pediatric populations. The absence of a standardized long COVID definition, along with its novelty in pediatric cases and symptom overlap with other conditions, contributed to discrepancies in our study. Similar challenges have been encountered when defining other postviral syndromes.

One major challenge involved patients with comorbidities, where it was often difficult to distinguish between new symptoms and worsening preexisting symptoms. In most CP+/CR− cases, clinicians acknowledged the presence of symptoms, but based on their clinical judgment, were more likely to attribute these symptoms to preexisting conditions rather than long COVID when comorbidities were present. This likely led to the misattribution of long COVID symptoms, particularly in patients with medical complexity. Additionally, non-COVID-19 respiratory infections, which are common in children and can present with symptoms similar to long COVID, contributed to disagreement. When the modified phenotype excluded symptoms associated with non-COVID-19 infections, performance improved.

Additional factors may have contributed to the disagreement between the chart review and the CP. First, chart review and EHR-based algorithms can be susceptible to biases, such as incomplete or missing data, inconsistent clinical documentation, and fragmented care across health systems. Some long COVID-related conditions lack unique medical codes. For example, an ICD code for POTS did not exist until October 2022. 32 Its symptom overlap and clinician unfamiliarity make accurately identifying it through EHR data difficult. 33

In addition, chart reviewers had access to unstructured clinical notes, which may have included attribution details not captured in the structured data used by the CP. Using advanced methods, such as machine learning and natural language processing, to incorporate unstructured data could improve the phenotype's sensitivity and specificity, especially for complex cases where symptoms are described in narrative form.

Additionally, variations in the time window for assessing postacute symptoms may have impacted performance. The CP assessed symptoms up to 180 days post-SARS-CoV-2 infection, which may be too narrow, as clinicians indicated that they may use a longer time window. While extending the time frame may improve identification, it may also result in the later onset of symptoms not being clearly attributable to an infection. The CP also excluded certain symptom domains, particularly mental health conditions like anxiety, due to inconsistent recording in structured data and challenges in distinguishing between biological and social determinants. Developing CPs that incorporate subphenotypes (e.g., psychological) will be important for capturing the diverse manifestations of long COVID.

### Strengths

A key strength of our study is the integration of both a CP and a clinician chart review. Prior research assessing long COVID CPs in children and adults has rarely incorporated chart review, 20 21 which limits the ability to qualitatively assess cases where the two methods disagreed. By including a chart review, we were able to examine these discrepancies in detail.

Another strength is our focus on pediatric patients, a group understudied in long COVID research. While long COVID has a lower incidence in children compared with adults, 4 current diagnostic tools may not be sensitive enough to detect all cases. Our study design, which used data-driven symptom clusters and diagnosis codes, identified patients with long COVID-like symptoms, even when the presentation is unclear. Although this made our phenotype more inclusive, it may still be less inclusive than a clinician's subjective assessment. Finally, our inclusion of patients from 16 healthcare systems improved the generalizability of findings.

### Limitations and Future Directions

Our study has limitations but also highlights areas for future work. One limitation is the potential bias against patients with preexisting chronic conditions, which may have influenced both the phenotype and the chart review. Clinicians may have attributed post-COVID-19 symptoms to these chronic conditions, reducing agreement with the phenotype. Additionally, differentiating chronic condition progression from COVID-related symptom worsening using EHR data was challenging and prevented the assessment of symptom exacerbation. Future research should consider cluster-specific washout windows to better identify worsening preexisting conditions.

Another limitation is our sampling strategy, which included edge cases and rare occurrences rather than random sampling. While this helped identify diverse long COVID symptoms, it may have limited generalizability and underestimated the phenotype's performance. Random sampling of CP-negative cases could have improved specificity, but it would have reduced the informativeness of negative cases. We also oversampled “probable” long COVID patients, who were those who did not receive a diagnosis but had symptoms suggestive of long COVID, to capture underrepresented presentations. This likely reduced sensitivity but provided insight into undiagnosed patients.

In addition, our requirement of at least two postacute visits may have biased the sample toward children with greater healthcare access, potentially excluding those with milder cases or limited access to follow-up care. This selection bias could have impacted the observed performance of the phenotype. Future research should evaluate the phenotype's performance in more nationally representative cohorts to assess its generalizability. Such efforts will be critical to ensure broader applicability and to promote equity in long COVID research. Finally, because each chart was adjudicated by a single clinician, we could not assess interrater reliability. Although we minimized variability through standardized protocols and reviewer training, some subjectivity likely remains. Future studies should include multiple reviewers for a subset of charts to assess interrater reliability.

## Conclusion

This study used a CP to identify children with long COVID using EHR data and compared its performance to clinician identification. Our findings highlight the complexity of identifying long COVID and underscore the need for a standardized definition, as emphasized by the National Academies of Sciences, Engineering, and Medicine (NASEM). 34 Although NASEM has proposed a broad working definition, continuous updates will be necessary as the understanding of this disorder, especially in children, evolves.

Future efforts should focus on integrating unstructured data and refining algorithms with clinical input. Incorporating the worsening of comorbidities into case definitions will also be critical. Ultimately, a validated CP will enable large-scale pediatric long COVID research, advancing both observational studies and clinical trials.

## Clinical Relevance Statement

Long COVID in children is characterized by a range of symptoms, including fatigue, headache, and more severe conditions like myocarditis and POTS. The lack of a standardized clinical definition makes diagnosis and research focused on this population challenging. We developed a rule-based CP using EHR data to improve the identification of pediatric long COVID. When compared with manual chart review, the algorithm showed moderate agreement in identifying long COVID. Discrepancies between the algorithm and chart review were primarily due to clinician reviewers attributing long COVID-like symptoms to prior or co-occurring medical conditions. This variation suggests that different frameworks are being used by clinicians to identify long COVID, complicating the ability to build a common research definition for large-scale analytics. These findings underscore the need for a standardized definition as accurate identification of long COVID is critical for timely treatment and improving outcomes for children.

## Multiple-Choice Questions

1. Which of the following was used as the primary data source for identifying pediatric long COVID cases in this study?
  - Patient self-reports and surveys.
  - Repository of electronic health record (EHR) data.
  - Genetic analysis of COVID-19 variants.
  - National vaccination registries.
  **Correct Answer** : The correct answer is option b. The data source for this study is the RECOVER PCORnet EHR database, which includes more than 9 million children from 40 hospital systems across the United States who were tested, diagnosed, or vaccinated for COVID-19 between 2019 and December 2022.
2. What method was used in the study to improve the identification of pediatric patients with long COVID?
  - Manual chart review only.
  - A rules-based computable phenotype approach.
  - An analysis of only symptoms related to fever and respiratory conditions.
  - A nationwide survey of COVID-19 survivors.
  **Correct Answer** : The correct answer is option b. The rules based CP takes into consideration the symptoms present in a patient post-COVID-19 infection, in addition to solely diagnosis codes specific to long COVID, to assign a level of certainty that the patient developed long COVID.
3. What was the main goal of the study mentioned in the text?
  - To determine the genetic basis of long COVID.
  - To assess the effectiveness of vaccines in preventing long COVID.
  - To improve the identification of pediatric long COVID using a computable phenotype approach.
  - To compare COVID-19 treatments across different age groups.
  **Correct Answer** : The correct answer is option c. Identifying children with long COVID is challenging due to inconsistency and heterogeneity of symptoms. By developing the rules-based CP we aim to improve identification of pediatric long COVID by considering new and persistent clusters of conditions post-COVID-19 infection.
